# Practical Recommendations Relevant to the Use of Resistance Training for COVID-19 Survivors

**DOI:** 10.3389/fphys.2021.637590

**Published:** 2021-03-03

**Authors:** Paulo Gentil, Claudio Andre Barbosa de Lira, Victor Coswig, Weimar Kunz Sebba Barroso, Priscila Valverde de Oliveira Vitorino, Rodrigo Ramirez-Campillo, Wagner Martins, Daniel Souza

**Affiliations:** ^1^College of Physical Education and Dance, Federal University of Goiás, Goiânia, Brazil; ^2^Hypertension League, Federal University of Goiás, Goiânia, Brazil; ^3^College of Physical Education, Federal University of Pará, Castanhal, Brazil; ^4^Social Sciences and Health School, Pontifical Catholic University of Goiás, Goiânia, Brazil; ^5^Laboratory of Human Performance, Quality of Life and Wellness Research Group, Department of Physical Activity Sciences, Universidad de Los Lagos, Osorno, Chile; ^6^Centro de Investigación en Fisiología del Ejercicio, Facultad de Ciencias, Universidad Mayor, Santiago, Chile; ^7^Physiotherapy College, University of Brasília, Brasília, Brazil

**Keywords:** resistance exercise, rehabilitation, strength training, pulmonary rehabilitation, cardiac rehabilitation, coronavirus

## Abstract

The novel coronavirus disease (COVID-19) has emerged at the end of 2019 and caused a global pandemic. The disease predominantly affects the respiratory system; however, there is evidence that it is a multisystem disease that also impacts the cardiovascular system. Although the long-term consequences of COVID-19 are not well-known, evidence from similar diseases alerts for the possibility of long-term impaired physical function and reduced quality of life, especially in those requiring critical care. Therefore, rehabilitation strategies are needed to improve outcomes in COVID-19 survivors. Among the possible strategies, resistance training (RT) might be particularly interesting, since it has been shown to increase functional capacity both in acute and chronic respiratory conditions and in cardiac patients. The present article aims to propose evidence-based and practical suggestions for RT prescription for people who have been diagnosed with COVID-19 with a special focus on immune, respiratory, and cardiovascular systems. Based on the current literature, we present RT as a possible safe and feasible activity that can be time-efficient and easy to be implemented in different settings.

## The Problem

The novel coronavirus disease (COVID-19) pandemic has posed a great threat to public health concern and safety (Wu et al., 2020; Zu et al., 2020). Caused by acute respiratory syndrome coronavirus 2 (or SARS-CoV-2), COVID-19 is characterized by respiratory distress and multisystem disease, which is frequently severe and might result in death (Kreutz et al., 2020). Many COVID-19 survivors who required critical care may develop psychological, physical, and cognitive impairments (Barker-Davies et al., 2020). There is evidence that coronaviruses may induce neurological impairments by invading the central nervous system and some patients may have symptoms like severe muscle pain (Li Y. C. et al., 2020). COVID results in relevant morbidity for 3–6 months (intermediate phase), and rehabilitation services and medical care might be needed for more than 12 months (chronic phase) (Barker-Davies et al., 2020).

Previous studies showed that survivors of acute respiratory diseases might have persistent functional disability and psychological symptoms for as much as 1 year after discharge (Herridge et al., 2003; Tansey et al., 2007), with most of them showing extrapulmonary conditions, with muscle wasting and weakness being most frequent (Herridge et al., 2003). Moreover, many COVID-19 patients will need to be on intensive care units, which is associated with symptoms like dyspnea, anxiety, depression, impaired physical function, and poor quality of life for up to 12 months after discharge (Oeyen et al., 2010; Denehy and Elliott, 2012; Jackson et al., 2012). Among them, physical function is one of the factors least likely to recover to normal values as it is heavily affected by critical illness (Gerth et al., 2019). The cardinal manifestations include limb muscle weakness, muscle atrophy, and impairments in deep tendon reflexes (Li Z. et al., 2020). Neuromuscular weakness in the intensive care units can prolong the patient’s mechanical ventilation time and hospitalization. Therefore, rehabilitation should commence in the critical care setting, since early exercise prevents neuromuscular complications and improves functional status in critical illness, being considered effective, safe, and feasible (Sosnowski et al., 2015; Barker-Davies et al., 2020). Moreover, rehabilitation programs starting within the post-acute phase (<30 days) seem to bring the most benefits (Barker-Davies et al., 2020).

Besides all the knowledge about intensive care management and recovery, there is a paucity of evidence-based recommendations regarding rehabilitation following COVID-19. Among the possible strategies for rehabilitating COVID-19 patients survivors, resistance training (RT) that conventionally consists of the voluntary muscle contractions against some kind of external resistance might be particularly interesting, since it has been shown to be a safe and feasible strategy to increase functional capacity in both acute and chronic respiratory conditions (Troosters et al., 2010; Liao et al., 2015; Li et al., 2019; Rice et al., 2020). Based on the current scientific evidence, RT can be safe, time-efficient, and easy to be implemented in almost anywhere and with minimal resources (Gentil et al., 2020b; Souza et al., 2020). Therefore, the present article aims to propose evidence-based and practical suggestions for the use of RT for people who have been diagnosed with COVID-19 during different phases of disease, with a special focus on immune, respiratory, and cardiovascular systems.

## Immune System

The immune system works through the coordinated functions of many cells to protect the organism against opportunistic infections (Pedersen and Hoffman-Goetz, 2000). Therefore, preserving or improving its function is important for people who were affected by COVID-19. There are evidences of either immune surveillance or immunodepression in response to exercise (Pedersen et al., 1998; Peake et al., 2017; Nieman and Wentz, 2019); however, the specific effects of RT on immune function have not being extensively studied (Freidenreich and Volek, 2012). Interestingly, people involved in endurance training are more commonly affected by immunodepression and illness (Nieman, 2007) when compared to strength and power sports (Alonso et al., 2010, 2012; Horn et al., 2010; Timpka et al., 2017), which might be a favorable point to RT (Natale et al., 2003; Gentil et al., 2020b). In general, the association between exercise and body immune defenses follows a J-shaped curve (Pedersen et al., 1998; Peake et al., 2017; Nieman and Wentz, 2019), improving with moderate amounts of physical exercise and decreasing with excessive or low amounts of exercise (Pedersen et al., 1998; Peake et al., 2017; Nieman and Wentz, 2019). This complex relation is negatively influenced by many factors, such as higher energy expenditure (Spence et al., 2007; Rama et al., 2013), increased exercise volume (Peters and Bateman, 1983; Gleeson et al., 2013; Siedlik et al., 2016), and metabolic stress (Pedersen and Hoffman-Goetz, 2000). In this sense, an acute bout of exercise might induce a suppressive effect on lymphocyte proliferative responses, with long-duration (longer than 1 h) and high-intensity exercise exhibiting a moderate suppressive effect (Siedlik et al., 2016).

A study by Davis et al. (1997) analyzed the effects of physical exercise on susceptibility to respiratory infection by using a murine model. The exercise design was composed of three groups: no exercise, moderate short-term exercise (30 min), and prolonged exercise to voluntary fatigue (2.5–3.5 h). According to the results, exercising to fatigue resulted in greater mortality rate (41%) than either no exercise or short-term moderate exercise. Although mortality rate tended to be lower after short-term moderate exercise (9%) than no exercise (16%), there was no significant difference between conditions. The results also showed a decrease in antiviral resistance after strenuous exercise within the lungs, in conjunction with increased susceptibility to respiratory infection *in vivo*. Although there is paucity of data linking the transitory immune suppression after strenuous exercise with chronic immune system impairment and subsequently infection risk (Nieman and Wentz, 2019), it is reasonable to suggest that exercise-induced immune suppression may impair the clearance of pathogen in acute illness COVID-19 patients. Therefore, even after the acute phase of the disease, physical exercise should ensure the adequate restoration of immune defense.

For these reasons, it might be advisable to avoid strenuous activities and adopt a reduced total training RT volume/duration (<45 min) to preserve immune function and decrease the risk of complications, particularly when the immune response is still compromised (Gleeson et al., 2013; Peake et al., 2017). With that in mind, low-volume RT should be recommended. Here, it is important to note that training sessions lasting a few minutes have been suggested to promote muscle strength and size gains in different populations (Fisher J. et al., 2017; Souza et al., 2020). From a practical standpoint, previous studies showed that untrained young and older adults can obtain many health benefits (e.g., increased functionality and cardiovascular improvements) from minimal dose RT protocols involving two sets of three to four basic exercises with a training frequency of one or two sessions per week (Fisher et al., 2014; de Barbalho et al., 2017; Seguro et al., 2019; Souza et al., 2019; Dias et al., 2020).

It is important to consider that rises in epinephrine, cortisol, and sympathetic modulation seem to be related to immunosuppression induced by exercise (Pedersen and Hoffman-Goetz, 2000; Nieman and Wentz, 2019). In this regard, previous studies have shown an association between elevated metabolic stress, cortisol levels, and immunosuppression in response to RT (Miles et al., 2003; Ramel et al., 2003; Krüger et al., 2011). Therefore, it might be interesting to avoid such responses in COVID-19 survivors under rehabilitation. According to previous studies, RT protocols with a few number of repetitions (≤6 repetitions) and long between-sets rest intervals (≥3 min) result in less pronounced increases in sympathetic activity, cortisol, and lactate levels (Kraemer et al., 1990; Smilios et al., 2003, 2007; Vale et al., 2018). Moreover, low-volume RT with few repetitions is less glycolytic (Knuiman et al., 2015). Therefore, it could prevent the concurrency for energy substrate and subsequent immunosuppression, since glucose is the main fuel of immune cells (Palmer et al., 2015).

Regarding time of the day, studies involving endurance activities showed that the acute increases in leukocytes were higher when exercise was performed during the night (6 PM) when compared to morning (9 AM), and it remained high for 1 h after exercise in a hot and humid weather (Boukelia et al., 2018). When comparing exercise during the morning and afternoon (9 AM vs. 4 PM) in a cold environment, Boukelia et al. (2017) found higher immune function and less pulmonary inflammation during afternoon exercise. We could not find specific studies with RT; however, it has been previously shown that plasma cortisol levels are increased during the morning (Hayes et al., 2010), which could suggest an impaired immune function. Therefore, the suggestion is to train in the afternoon or early night.

The basis of COVID-19 pathogenesis is associated with a delayed antiviral response followed by an immunological overreaction that results in an excessive proinflammatory state (Castelli et al., 2020). The levels of systemic inflammation might explain the severity of the disease, with the most affected patients presenting higher serum levels of proinflammatory cytokine, as well as reduced T lymphocytes count (Chen et al., 2020). Regulatory T lymphocyte (Treg) is also reduced in severely ill patients and seems to play an important role in COVID-19 pathogenesis, since it is associated with controlling autoimmune and proinflammatory response (Gladstone et al., 2020; Stephen-Victor et al., 2020). In this context, RT may contribute to control proinflammatory state (Chupel et al., 2017; Santiago et al., 2018; Lammers et al., 2020). Despite the fact that studies investigating the effect of RT on Treg cells are scarce (Dorneles et al., 2020), a previous study in murine model showed that RT can upregulate this immune marker (Souza et al., 2017). Moreover, regular practice of RT increases the levels of interleukin-10, an anti-inflammatory cytokine that is mainly produced by Treg cells (Chupel et al., 2017; Lammers et al., 2020).

## Respiratory System

The high levels of proinflammation mediators and histopathological changes in the lungs in response to SARS-CoV-2 might induce apoptosis in pulmonary endothelial and epithelial cells, leading to impaired respiratory function such as acute respiratory distress (Castelli et al., 2020). Additionally, persistent proinflammatory state in severe COVID-19 patients is associated with fibroblast proliferation in the alveolar septum, resulting in pulmonary interstitial fibrosis (Zhang et al., 2020). Pulmonary diseases are commonly associated with loss of muscle mass and function (Steiner, 2007; Bone et al., 2017). The analysis of previous outbreaks of severe acute respiratory syndrome (SARS) revealed that 6–20% of the patients showed mild or moderate restrictive lung function consistent with muscle weakness 6–8 weeks after hospital discharge (Chan et al., 2003). This seems to persist for an even longer period as persistent pulmonary function impairment was present in 37% of the patients after recovery from SARS and their health status was also significantly worse compared with healthy subjects (Ong et al., 2005). Results from a cohort study showed significant impairment in lung capacity in 23.7% of SARS survivors 1 year after illness onset (Hui et al., 2005). Moreover, health status and exercise capacity were remarkably lower than those found in the normal population (Hui et al., 2005).

Previous studies showed that, in people with pulmonary diseases, low muscle strength is associated with physical inactivity (Osthoff et al., 2013) and is an independent predictor of morbidity and mortality independent of the degree of respiratory limitation (Swallow et al., 2007). Consequently, the key target in rehabilitation for pulmonary diseases should be improving locomotor muscle structure and function, as exercise results in reduced benefits on exertional ventilation, operating lung volumes, and respiratory muscle performance (Marillier et al., 2020). Moreover, the performance of physical exercise is advised as adjuvant non-pharmacological treatment during pulmonary fibrosis rehabilitation (Spruit et al., 2009).

RT has been suggested as an successful strategy for pulmonary rehabilitation, either performed alone or in conjunction with aerobic training, since it brings important increases in functional capacity (Liao et al., 2015; José and Dal Corso, 2016; Li et al., 2019). It is also important to highlight that exercise training during hospitalization due to acute respiratory conditions seems to bring important health and functional benefits, is well tolerated, and the adverse events are infrequent (Troosters et al., 2010; Rice et al., 2020). RT can be successfully performed as a stand-alone exercise strategy, without increasing adverse events in chronic obstructive pulmonary disease patients under pulmonary rehabilitation (Liao et al., 2015).

Considering that most people infected with SARS-CoV-2 could experience breathing difficulties, it is recommended to control the respiratory responses to exercise. One advantage of RT is that it might promote less cardiorespiratory stress (i.e., oxygen consumption and pulmonary ventilation) than aerobic exercise, even during maximal exercise testing (Houchen-Wolloff et al., 2014; Garnacho-Castaño et al., 2015; Albesa-Albiol et al., 2019). The manipulation of RT variables might further reduce the respiratory stress. Pulmonary ventilation and oxygen consumption increase with increased volume/duration (Haddock and Wilkin, 2006; Mookerjee et al., 2016; Garnacho-Castaño et al., 2018), lower rest intervals (Ratamess et al., 2007; Farinatti and Castinheiras Net, 2011), higher movement velocities (Mazzetti et al., 2011; Mukaimoto and Ohno, 2012; Buitrago et al., 2014), and higher number of repetitions (Scott et al., 2011; Ratamess et al., 2014). Therefore, training with lower number of repetitions, higher interval between sets, and controlled movement velocity might be recommended (Buitrago et al., 2013).

## Cardiovascular System

Similar to other coronavirus infections, COVID-19 is associated with cardiac complications, especially arrhythmias, heart failure, and myocardial injury (Kochi et al., 2020; Madjid et al., 2020; Wang et al., 2020). Acute cardiac injury is higher in those with increased mortality, with severe disease, and requiring ventilatory support (Kochi et al., 2020; Madjid et al., 2020). Cardiac complications have been suggested to be multifactorial. It may be caused by hypoxia, viral myocardial injury, hypotension, ACE2-receptor downregulation, drug toxicity, or elevated systemic inflammation (Kochi et al., 2020). The proinflammatory mediators associated with COVID-19 can result in vascular inflammation, myocarditis, and arrhythmic complications (Kochi et al., 2020; Madjid et al., 2020). Another complication regarding cardiovascular system is the increased risk of thromboembolism as a consequence of coagulopathy and endothelial vascular dysfunction in critical illness COVID-19 patients (Goshua et al., 2020).

Patients diagnosed with COVID-19 should be fully assessed and, if necessary, additional investigations may include resting electrocardiogram (ECG), blood exams, 24 h ECG, cardiopulmonary, echocardiogram, cardiovascular magnetic resonance imaging, and exercise testing with the involvement of a cardiologist (Barker-Davies et al., 2020). In case of myocarditis, a period of 3–6 months of complete rest from strenuous exercise might be necessary, depending on the clinical severity illness duration (Pelliccia et al., 2019; Schellhorn et al., 2020). After returning, it is advisable to conduct periodic reassessment in the first 2 years due to an increased risk of silent clinical progression (Pelliccia et al., 2019).

RT has been shown to be safe and effective for several cardiac patients from different cardiac diseases and has been recommended as a core component of cardiac rehabilitation for many decades (McKelvie and McCartney, 1990; Verrill et al., 1992; Yamamoto et al., 2016). Some studies suggested that RT might be even safer than aerobic exercise, since it results in less myocardial stress and reduced hemodynamic responses in patients with heart diseases like controlled heart failure (Karlsdottir et al., 2002; Levinger et al., 2005), coronary arterial disease (Karlsdottir et al., 2002), and ischemic cardiomyopathy (McKelvie et al., 1995) and in patients in cardiac rehabilitation after myocardial infarction and percutaneous coronary intervention (Adams et al., 2010). Moreover, RT leads to improvements in cardiac autonomic control of diseased individuals (Bhati et al., 2019).

Cardiovascular stress might be more related to the duration of the exercise than with the load used, granting the use of higher loads and a lower number of repetitions. In this regard, Lamotte et al. (2005) reported higher levels of blood pressure and heart rate in response to RT using lower external loads and higher repetitions [four sets of 17 repetitions at 40% of the one-repetition maximum strength (1RM)] when compared with higher external loads and lower repetitions (four sets of 10 repetitions at 70% of 1RM) in 14 patients who participated in a rehabilitation program (e.g., bypass surgery, percutaneous coronary angioplasty, or valvular surgery). Similarly, Gjøvaag et al. (2016) reported higher levels of blood pressure and heart rate in patients with coronary arterial disease after performing 15RM with lower external loads than performing 4RM with higher external loads. Regarding autonomic modulation, Vale et al. (2018) showed that hypertensive women training with lower repetitions and higher external loads (6RM) showed less sympathetic activation and higher parasympathetic activation when compared to training with lower external loads and more repetitions (15RM). Therefore, in order to reduce cardiovascular stress during exercise, the recommended RT program should involve lower number of repetitions regardless of the load used.

One important feature in previous studies is that blood pressure and heart rate progressively increase over the sets, especially when the rest between sets is shorter (Gotshall et al., 1999; Lamotte et al., 2005; Gjøvaag et al., 2016). This suggests that one should consider performing a lower number of sets (one or two) and using higher rest between sets (≥3 min). Other additional strategies to reduce cardiovascular stress is to give short pauses (i.e., 5 s) in the middle of the sets (da Silva et al., 2007; Rúa-Alonso et al., 2020), avoid performing repetitions until muscle failure (MacDougall et al., 1992), and exercise during the afternoon, since cardiac reactivity is lower (Jones et al., 2006; Boukelia et al., 2018) and there is a better blood pressure control (Jones et al., 2008) at this period of the day.

## Practical Recommendations

RT might be performed in many settings, including acute hospitalization and rehabilitation scenarios. Previous studies have shown that RT performed during intensive care units might bring important benefits either alone (Morris et al., 2016; Barbalho et al., 2019; Veldema et al., 2019) or combined with other activities (Eggmann et al., 2018). Interestingly, the benefits of RT in intensive care unit patients have been reported even in the presence of mechanical ventilation (Eggmann et al., 2018).

**FIGURE 1:**
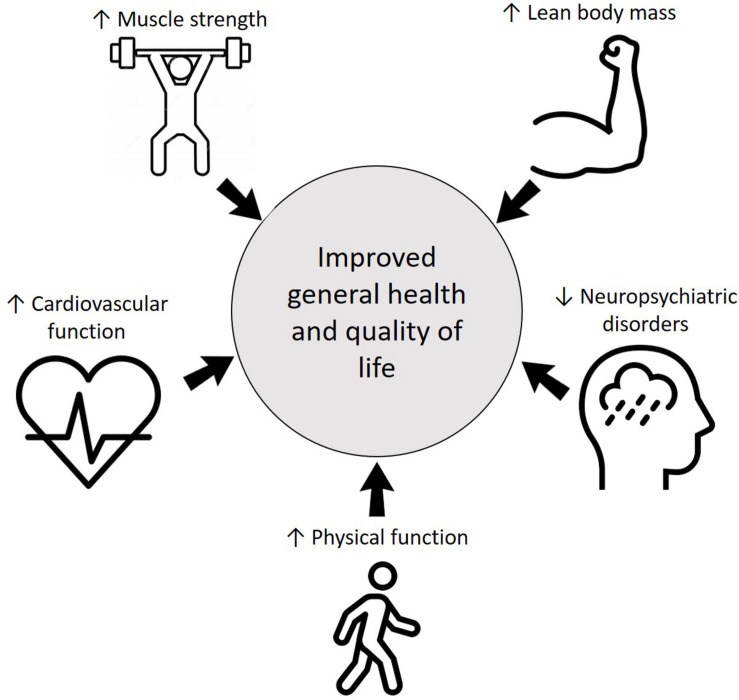
Multi-system benefits of resistance training.

Another important concern with COVID-19 is the neuropsychiatric sequalae. In addition to pandemic-associated psychological distress, the direct and indirect effects of the coronavirus on the human central nervous system might be related to neuropsychiatric disorders such mood changes, sleep disorders, depression, and anxiety (Khatoon et al., 2020; Steardo et al., 2020; Troyer et al., 2020). Studies investigating COVID-19 patients found a high level of post-traumatic stress and depressive symptoms in comparison with non-infected people (Vindegaard and Eriksen Benros, 2020). In this regard, there are consistent evidence that RT is associated with improvements in depression (Gordon et al., 2018), anxiety (Gordon et al., 2017), and sleep disorders (Kovacevic et al., 2018), including patients with chronic diseases (Ferreira et al., 2020) and during rehabilitation (McCartney, 1998; Vincent and Vincent, 2012; Chan and Cheema, 2016; Andrade et al., 2018; Seguro et al., 2019). The potential benefits of RT for COVID-19 patients are illustrated in Figure 1.

RT programs commonly involve many exercises with the addition of isolated exercises for specific muscles, which might be too time-consuming. However, multi-joint exercises seem to be sufficient to improve muscle strength and hypertrophy in the muscles involved in the exercises (Gentil et al., 2015, 2017b; Paoli et al., 2017; Barbalho et al.,2020a,b) and there is no additional benefits in using single-joint exercises (Gentil et al., 2013; de França et al., 2015; Barbalho et al., 2020b). This allows the use of multi-joint exercises combined with low-volume programs, increasing feasibility and safety for most of the patients affected by COVID-19, hospitalized or not, including individuals with cardiometabolic diseases and frail elderly. Patients with COVID-19 that present severe body aches, sore throat, shortness of breath, chest pain, general fatigue, cough, or fever should avoid exercises between 2 and 3 weeks after the cessation of these symptoms. It is also recommended to avoid prolonged exhaustive or high-intensity exercise. These current restrictions to RT practice could be reviewed after cessation of the symptoms. COVID-19 patients that are asymptomatic should continue to exercise, as they would do normally. A pulmonary rehabilitation approach should be combined in the case on return from mild/moderate COVID-19 illness (Barker-Davies et al., 2020).

RT using non-traditional equipment such as elastic devices, which are low cost and portable, and can be performed in almost anywhere, might contribute to increase the possibilities for RT performance in many different settings, including intensive care units. Previous studies reported that RT using elastic bands or tubes resulted in similar muscle activation and mechanical stress (Aboodarda et al., 2011, 2016), strength gains (Martins et al., 2013), and improvements in functional capacity (Colado et al., 2010; Souza et al., 2019) when compared to traditional RT. Furthermore, RT might also be performed using body weight exercises as it promotes gains in muscle strength, hypertrophy, and body composition similar to traditional RT for many different populations, like middle-aged people with non-alcoholic fat liver disease (Takahashi et al., 2015, 2017), elderly people (Tsuzuku et al., 2017), and even young trained practitioners (Calatayud et al., 2015; Kikuchi and Nakazato, 2017).

Another possible limitation in rehabilitation settings is the belief that RT has to be performed with moderate to high loads (ACSM, 2009; Kraemer et al., 2002), as it is commonly suggested that it would be necessary to use loads ≥60% of 1RM for optimal gains in strength and muscle mass (McDonagh and Davies, 1984; ACSM, 2009). However, previous studies have shown that low external load RT might bring increases in muscle fitness and hypertrophy that are similar to conventional approaches, when effort is high (Fisher J. P. et al., 2017; Steele et al., 2019). Previous studies in both trained (Morton et al., 2016) and untrained people (Mitchell et al., 2012; Assunção et al., 2016) reported that RT with low external load resulted in similar increase in muscle strength and hypertrophy when compared to high external load. This is particularly evident when the strength tests not similar to the situations trained (Fisher J. P. et al., 2017). The caveats for using low external load are that it would require a higher number of repetitions and longer exercise times, which can result in more negative impact on the immune system and a higher stress on respiratory and cardiovascular systems, as suggested above. Therefore, the cost–benefit of such adaptations might be analyzed individually.

Significant physiological stimulus can also be obtained with maximal or near-maximal voluntary muscle contractions performed without external load. In this regard, previous studies reported high levels of muscle activation when performing RT with the intention to maximally contract the muscles and no external load (Gentil et al., 2017a; Alves et al., 2020). A previous study reported equivalent gains in arm muscle hypertrophy after traditional and no external load RT in young men and women, using a contralateral training design (Counts et al., 2016). Positive outcomes in terms of hypertrophy and functionality have also been reported in intensive care units patients (Barbalho et al., 2019).

Particularly in aging people, the performance of high-velocity RT might be considered as an alternative strategy when the performance of high or low external load RT with high effort is not possible or recommended (Fragala et al., 2019). High-velocity RT may provide superior increases on functional capacity in comparison with conventional RT (Bottaro et al., 2007; Nogueira et al., 2009; Ramírez-Campillo et al., 2014). A previous study suggested that high-velocity RT might be a feasible and safe strategy to revert or prevent functional decline during acute hospitalization (Martínez-Velilla et al., 2019). Thus, the performance of few repetitions using high-velocity concentric muscle action combined with long rest intervals and/or intra-set short pauses could provide significant gains on functionality while preventing higher cardiovascular stress (Lamotte et al., 2010; Dias et al., 2020). Considering that the use of light to moderate loads (e.g., 30–60% of 1RM) are recommended to optimize muscle power (Fragala et al., 2019), this might be easily achieved with small implements such light dumbbells or elastic devices. Therefore, equipment and implements should not be a barrier to implement RT programs during COVID-19 rehabilitation.

RT progression should be based on individual analysis, considering performance parameters and clinical symptoms. Initially, it is recommended that progression should be performed through increases in load, since higher number of sets and repetitions and lower rest intervals might impose unwanted risks. Therefore, the recommendation is to establish a repetition margin (i.e., 4–6RM) and increase load when the participant reaches the upper limit. When the patient reaches pre-COVID physical capacity, it would be interesting to re-examine for the possibility of restoring normal routine (Phelan et al., 2020).

## Final Considerations

It is important to observe some general precautions for returning to exercise post-COVID-19, like monitoring temperature before training, starting with a muscle strengthening program prior to cardiovascular work, keeping social distancing, observing hygiene, adequate ventilation, and the use of masks when necessary (So et al., 2004; Gentil et al., 2020a). Another relevant point is the need to carefully evaluate clinical status and supervise patients that have been diagnosed with COVID-19, especially people with cardiac injuries (Barker-Davies et al., 2020), highlighting the need of a multidisciplinary approach. A subclinical myocardial injury may be present after clinical recovery from mild infections, even without cardiac symptoms or hospital admission. While the present article addresses RT for rehabilitation purposes, medical clearance is required. Therefore, a medical evaluation is recommended to exclude subclinical diseases before resuming high-intensity training or competition, eventually with exams such as transthoracic echocardiogram, maximal exercise testing, and 24 h Holter monitoring (Dores and Cardim, 2020; Wilson et al., 2020).

Considering the negligible chance of cardiac sequelae after asymptomatic infection or local symptoms of COVID-19, it is not necessary to perform pre-participation screening if a critical evaluation of signs and symptoms is negative and shows a complete recovery (Verwoert et al., 2020; Wilson et al., 2020). However, a pre-participation screening and cardiologist consultation may be considered for specific groups, including, but not limited to, people with pre-existent cardiovascular disease, elite athletes, and those with impaired recovery of exercise capacity.

For those with regional or symptoms not requiring hospitalization, it is strongly recommended to perform a pre-participation screening that includes physical examination, critical evaluation of symptoms, and a 12-lead ECG (Verwoert et al., 2020; Wilson et al., 2020). A cardiologist experienced in reading athletes’ ECG should be consulted to differentiate between ECG changes due to exercise adaptation and ECG abnormalities suggestive of cardiac disease. This is necessary because 12-lead ECG is not the gold standard for the detection of myocarditis. It is also recommended to use cardiac biomarkers to detect myocarditis (Verwoert et al., 2020; Wilson et al., 2020). However, caution should be taken when using this strategy because most people do not have previously documented baseline measurements to compare with, and exercise might elevate the levels of these biomarkers, without clear-cut clinical implications (Verwoert et al., 2020). RT may be done after myocarditis if serum biomarkers of myocardial injury and left ventricular systolic function are normal and if 24 h ECG monitoring or exercise testing rules out relevant arrhythmias (Barker-Davies et al., 2020).

**FIGURE 2:**
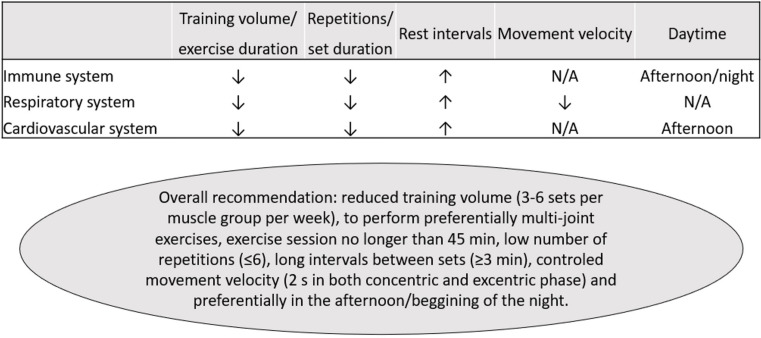
Practical recommendations for resistance training in COVID-19 survivors. ↑, higher; ↓, lower. N/A, not available.

It is worthy to note that most of these screening recommendations refer to competitive athletes and high intense activities (Dores and Cardim, 2020; Verwoert et al., 2020; Wilson et al., 2020). Therefore, the specific limitations for performing RT should be individually analyzed and consider the specificities of each protocol. In this context, RT might be designed to be especially safe for people who have been diagnosed with COVID-19, in different stages of disease and recovery, by decreasing the risk of immunosuppression and reducing respiratory stress and cardiovascular risk. Interestingly, when combining the evidence in immune, pulmonary, and cardiovascular systems, the use of low volume/duration approaches and the manipulation of training variables (moderate to high loads, short set duration, low number of sets, exercise choice, high rest intervals, and/or intra-set rest) might be particularly safe (Figure 2). RT might be also convenient as it can be performed with different implements (traditional machines, elastic devices, body weight exercises, or with no external load) and settings (in-hospital, exercise facilities, or home based), increasing its feasibility.

Finally, RT as an approach of the rehabilitation treatment should be individualized according to the patient’s need, taking into consideration their comorbidities, symptoms of dyspnea, and psychological distress.

## Data Availability Statement

The raw data supporting the conclusions of this article will be made available by the authors, without undue reservation.

## Author Contributions

PG and DS: conceptualization and writing the first draft. PG, CL, VC, WB, PV, RR-C, WM, and DS: writing, review, and editing. All authors contributed to the article and approved the submitted version.

## Conflict of Interest

The authors declare that the research was conducted in the absence of any commercial or financial relationships that could be construed as a potential conflict of interest. The handling editor declared a past co-authorship with one of the authors PG.
