# Supplementary material for: Practical Recommendations Relevant to the Use of Resistance Training for COVID-19 Survivors
Source: Front Physiol. 2021 Mar 3;12:637590. doi: 10.3389/fphys.2021.637590 (PMC7966515; doi:10.3389/fphys.2021.637590)
Supplement: Supplementary file 1 [file Table_1.docx]

Table 1: summary of the studies included.

| Study | Protocol | Results |
| --- | --- | --- |
| Immune system |  |  |
| Alonso et al., 2010 | Prospective recording of illnesses in 1979 athletes. | The highest incidence of illness was found in athletes involved inlong distance walking and the lowest was for throwing,jumpingand running up to 1500 m. |
| Alonso et al., 2012 | Prospective recording of illnesses in 1851 athletes. | The highest incidences of illness were found in long-distance walking events. |
| Boukelia et al., 2017 | 8 runnersperforming a 10kmtime trial, at 9AM and 4PM at 6 °C | Higher values for bloodneutrophil and lymphocyte count at4PM. P-CC16 was higher at the pre- and post-trial at 9AM. No difference in lung function. |
| Boukelia et al., 2018 | 13 runnersperforming a 10kmtime trial, at 9AM and 6PM at 28 °C | Higher values for total WBC, neutrophil and lymphocyteconcentrations at 6PM. More pronounced response in IL-6, HSP70, WBC, neutrophil, lymphocyte, and CC16 at 6PM. |
| Chupel et al., 2017; | 33 older women performed RT or control for 28 weeks | IL-10,hemoglobin, mean cell volume and mean cell hemoglobin concentration increased in RT group. , while leukocyte and lymphocyte counts decreased. TNF-α and CRP increased in the control group. |
| Davis et al., 1997 | Rats were inhaled with herpes simplex type 1 virus and were studied 3 weeks after 30 minmoderate exercise, 2.5–3.5hstrenuous exercise or control. | Mortality and morbidity were greater after prolonged exercise (41% and 50%) than moderate exercise (9% and 13%) and control (16% and 25%). |
| Gleeson et al., 2013 | Analysis of immune function and sickness of 75 athletes and training volume for 4 months. | Training ≥11h/week resulted in higher IL-2, IL-4 and IL-10 productionby antigen-stimulated whole blood culture. Training ≥7h/week was associated with more than twofold increases in of URTI. |
| Horn et al., 2010 | Analysisof blood tests of 3,679athletes | Lower WBC and neutrophil counts in aerobic sports. Positive correlation between thesports’ aerobic–anaerobic score with neutrophil and WBC counts |
| Krüger et al., 2011 | 15 young men performing2 RT protocols with the same number of repetitions and different loads (60 vs 75% 1RM). Venous blood was taken before, immediately after, 3 h after, and 24 h after exercise. | Significant increase of lymphocyte apoptosis 3 h after 75% 1RM. Blood lactate, IL-6, CRP, and cortisol increased 3h after 75% 1RM. Significant correlation between the increase of apoptosis and cortisol levels 3h after exercise. |
| Lammers et al., 2020 | 31 hypertensive and 28 normotensive middle-aged women performed RT or control for 24 weeks | RT reduced IL-6, CRP, ATP levels and NTPDase and adenosine deaminase activities in hypertensive group. RT increased IL-10 levels in hypertensive group. |
| Miles et al., 2003 | 64 young women performed RT for lower and upper or for upper body alone for 24 weeks. Blood samples were collected pre- and post RT (6x10 repetitions at 75% 1RM in the squat)before and after 3 and 6 months. | Lymphocyte recruitment and proliferation following RT did not differ between training groups at any time. Exercise-induced increases inNK, CD4+, CD8+ and B lymphocyte concentrations were greater in those with greater lactate responses. |
| Natale et al., 2003 | 8 young men performing 5 minutes of cycleergometry exercise at 90-97%VO2max, 2 hours of cycleergometry exercise at 60-65% VO2max, circuit RT at 60-70% 1RM or control | All exercise types provoked leukocytosis that persisted for 3 hours. Leukocytosis was higher after prolonged aerobic aerobic exercise than hgher intensity. RT had the lowest leukocytosis.  The CD4+/ CD8+ ratio was decreased significantly immediately following all three types of exercise; howeverthe change was smaller for RT.  All exercise types induced increases in circulating NK cell counts immediately after exercise. The rise was greatest for higher intensity aerobic exercise, and lower for RT. However, all values returned to baseline after 3h.  The decreases in CD3+CD16+/56+count 3 hours after both aerobic exercises were greater than for RT. Higher intensity aerobic exercise B-cell counts immediately after exercise and RT induced a significant increase 3 hours after exercise. |
| Peters and Bateman, 1983; | Prospective study with 150 runners who took part in a 56km marathon | Symptoms of URTI occurred in 33.3% of runners compared with 15.3% of controls, and were most common in those who achieved the faster race times |
| Rama et al., 2013 | 19 swimmersand 11 non-athlete controls monitored for occurrence of URTI and circulating NK cells over awinter swimming season | In swimmers URTI were more common in periods of elevated training volume, while no URTI were reported atequivalent time points in non-athletes. Athletes showed decreases in the percentage and absolute counts of circulating NK cells in periods of increased training volume. |
| Ramel et al., 2003 | 7 resistance-trained and 10 non-resistance-trained malesperforming submaximal RT at 75% 1RM.Blood samples were taken before, during, immediately after, and 30, 60 and 120 min after exercise | Total leukocytes, monocytes and neutrophils increasedduring and 2h after RT. Lymphocytes increased during RT. T-helpercells returned to resting values after exercise, and natural killer cells and T-suppressor cells decreased belowresting values. CD4/CD8 ratio decreased during exercise but increased during recovery. Trained participants tended to have lower T-helper cell counts before, during and immediately after RT anda lower CD4/CD8 ratio during recovery. Plasma cortisol correlatedpositively with leukocytes during exercise, but negatively with T-helper cells 30 and 60 minafter RT |
| Santiago et al., 2018; | 19 older women performed RT or control for 8 weeks | RT decreasedIL-6, TNF-alpha and CRP levels. |
| Siedlik et al., 2016 | Meta-analysis with 24 articles involving the response of lymphocytes to acute exercise | Suppression of proliferative capacity following acute exercise in general. Exercise sessions longer than one hour have a greater suppressive effect regardless of exercise intensity |
| Souza et al., 2017 | Mice with autoimmune-induced encephalomyelitis performed RT, endurance training or control for 4 weeks | Increases on IL-10 concentration and regulatory T cells population after a RT session |
| Spence et al., 2007 | Prospective study 32 elite, 31 recreationally competitive triathletes and cyclists and 20 sedentary control for 5 months training and competitive period | Illness incidence was higher in elite athletes and sedentary controls than recreationally competitive athletes |
| Timpka et al., 2017 | Prospective study involving 957 high-levelathletes in pre-competitive and competitive period | Endurance athletes were more likely to sustain an illness during competition period than power/velocity athletes |
| Respiratory system |  |  |
| Albesa-Albiol et al., 2019 | 18 men performing aRT and cycle ergometer test at lactate threshold intensity | Cycle ergometer test induced a greater cardiorespiratory response in comparison with RT. Ventilatory efficiency was similar between modalities |
| Buitrago et al., 2013 | 10 men performing 4 RTprotocols until exhaustion: strength endurance, hypertrophy, maximum strength and high-speed endurance | Mean VO2 was higher during high-speed endurance in comparison with other programs. Blood lactate concentration was higher after high-speed endurance in comparison with maximum strength. VO2 was higher during strength endurance than hypertrophy and maximum strength |
| Buitrago et al., 2014 | 10 resistance trained men performing RT at 55%, 70% and 85% of 1RM using different velocities | An increase in mean concentric power induced a higher aerobic energy turnover rate |
| Farinatti and Castinheiras Net, 2011 | 10 men performing 4 RT protocols that consisted in5x 10 at 15RMload different RT exercises (leg pressor chest fly)and 1 and 3 min rest interval | 1min intervals induced higher VO2 during leg press in comparison with 3min rest intervals and chest fly. |
| Garnacho-Castaño et al., 2015 | 18 resistance trained subjectsperforming aRT and cycle ergometer test at lactate threshold intensity | Cycle ergometer test induced a greater cardiorespiratory response in comparison with RT.Mechanical fatigue was observed only in RT |
| Garnacho-Castaño et al., 2018 | 21 resistance trained subjects performingRT and cycle ergometer test at constant load | Cycle ergometer test induced a greater VO2 and heart hate response in comparison with RT. No difference for blood lactate concentration |
| Haddock and Wilkin,2006 | 15 resistance trained women performed RT with 1 and 3 sets | Higher volume RT induced greater increases in VO2 |
| Houchen-Wolloff et al., 2014 | People with COPD and health controls performing RT session (5 sets of maximal isokinetic knee extension) | There is no difference between COPD patients and health controls for VO2 and peak ventilation achieved during exercise |
| José and Dal Corso, 2016 | 49 hospitalized patients with pneumonia performed physical exercise or standard pulmonary rehabilitation for 8 days | Improvements in functionality were greater after inpatient physical exercise compared with standard pulmonary rehabilitation. Physical exercise group achieve significant improvements in muscle strength, dyspnea, and quality of life |
| Li et al., 2019 | Meta-analysis with 11 articles investigating the effect of RT on exercise capacity in COPD patients | RT is effective to improve functional exercise capacity, endurance, and peak exercise capacity in COPD patients |
| Liao et al., 2015 | Meta-analysis with 18 articles investigating the effect of RT on COPD patients | RT is effective to improve functional exercise capacity, dyspnea, pulmonary function in COPD patients |
| Mazzetti et al., 2011 | 7 trained and 7 untrained performing RT with 3 different velocities (2 s for both muscle actions; 1 s for both muscle actions; and 2 s for eccentric followed by concentric using maximal velocity) | VO2 during and after RT performed withmaximal velocity was higher when compared with RT performed with controlled velocity |
| Mookerjee et al., 2016 | 12 healthy subjects performing RT with 1 and 3 sets. Metabolic and cardiorespiratory data were recorded over the entire exercise session and during 5 minutes of recovery | HR, respiratory rate, VO2, respiratory exchange ratio, and minute ventilation values were significantly higher during the 3 sets than 1 set protocol. |
| Mukaimoto and Ohno, 2012 | 11 healthy men performing3 circuit RT protocols: 50%1RMand 4s for each of lifting and lowering phases; 80%1RM and 1s each phase; and 50%1RM and 1s each phase.VO2 was monitored continuously during exercise and for 180 min after exercise. | Average VO2 throughout the session was significantly higher with 50 and 80%1RM with faster movement than 50%1RM with slow movement. However, total VO2 was significantly greater for 50%1RM and slow movement. |
| Ratamess et al., 2007 | 8 trained men performing10 RT protocols [5 sets at 75 or 85%1RM for 10 or 5 reps, 30 s, 1, 2, 3 and 5 min or rest)]. VO2 was measured during exercise and for 30 min post exercise. | Mean VO2 and ventilation were progressively higher as rest interval was shortened. VO2 area under the was higher for 10 vs 5 reps. VO2 after 30s was higher than 2-, 3-, and 5-min and 1-min was higher than 5-min during 5 reps. Fatigue rate was correlated to all metabolic variables. |
| Scott et al., 2011 | 13 healthy men evaluated during one set of RT performed with 37, 46, 56, 70, 80 and 90%. | Lower loads resulted in higher VO2 |
| Swallow et al., 2007 | Retrospective study with 184 COPD patients | Mortality risk increased with increasing age and with reducing knee extensor strength. |
| Troosters et al., 2010 | 40 COPD patients divided in control and RT (3x8RM) during hospitalization (~7 days). Follow up of 30 days | RT group had higher quadriceps function, 6 min walking test and improved anabolic/catabolic balance. |
| Cardiovascular system |  |  |
| Adams et al., 2010 | 21 cardiac rehabilitation patients were evaluated during walking (RPE 11 in the 6-20 Bog Scale) and RT (3-5x10-12 at 50%1RM) | HR, SBP and RPP were higher during treadmill than RT |
| Boukelia et al., 2018 | 13 runnersperforming a 10kmtime trial run, at 9AM and 6PM at 28 °C | Higher HR response at 9AM |
| da Silva et al., 2007 | 12 older women performing RT (3x10RM) continuously or with 5 or 10s break between the 5^th^ and 6^th^ repetitions | Blood lactate, HR and RPP were significantly after the continuous protocols. |
| Gjøvaag et al., 2016 | 15 coronary patients during two RT protocols: 3x4RM or 3x15RM | HR, SBP and DBP higher during 15RM than 4RM SBP and DBP. |
| Gotshall et al., 1999 | 7 trained men were evaluated during RT involving 3x10RM | SBP increased over the repetitions and over the sets reaching 293mmHg |
| Jones et al., 2006 | 440 hypertensive patients accompanied for 24-hours | The highest reactivity of SBP to physical activity was observed between 8 and 10AM. Between 10AM and 12PM, BP reactivity then decreased and showed a secondary rise in the early afternoon. |
| Jones et al., 2008 | 12 healthy men evaluated for 90min after 30min of cycling at 70VO2peak at 8AM and 4PM | MBP and total peripheral resistance reduced following exercise 4PM, but increased when exercise was performed at 8AM. |
| Karlsdottir et al., 2002 | 12 healthy volunteers, 12 patients with stable coronary artery disease, and 12 patients with stable congestive heart failure were evaluated during cycling at 90% of ventilatory threshold, RT (10 reps at 60-70% 1RM) | Similar cardiovascular responses cycling and RT. The patients with CHF reported moderate muscle tiredness, and three patients experienced moderate shortness of breath during steady-state cycling |
| Lammote et al., 2010 | 17 male coronary patients performing 3x10 at 75%1RM at slow, moderate or fast velocity and also with 30, 60, 90 or 120s intervals. | Increases in HR and SBP over the sets. HR and SBP were higher with shorter rest intervals. |
| Lamotte et al., 2005 | 14 cardiac rehabilitation patients during two RT protocols: 4x17 at 40%1RM and 4x10 at 70%1RM | HR and SBP were higher during 4x17. Peak SBP increased from set 1 to set 3 and 4 during both protocols low and high intensity resistance training. |
| Levinger et al., 2005 | 15 men with chronic heart failure performed 8w of RT and were tested during Balke incremental tests (treadmill) and 1RM tests | RT increased walking time and peak VO2. Quality of life significantly increased. |
| MacDougall et al., 1992 | 31 healthy men were evaluated during different RT protocols | Exercise with the same relative intensity produced similar elevations in SBP independent of muscle action, muscle size, absolute load. Fatigue was associated with increased in SBP |
| McKelvie et al., 1995 | 10 men with congestive heart failure were tested during 3 situations: control, 5min of cycling at 70%PPO, 2x10 at 70%1RM | SBP increased similarly after cycling and RT. DBP increased more during RT. HR, cardiac output and stroke volumeincreased more during cycling. |
| Rúa-Alonso et al., 2020 | 32 healthy people (23 men and 9 women) were evaluated before and after 3 situations: control, RT performed with 4x10 (2min rest) and 8x5 (51s rest) | 4x10 induced greater reductions on cardiac parasympathetic modulation than 8x5 and control. 4x10 caused a higher lactate production and fatigue than 8x5. |
| Vale et al., 2018 | 15 postmenopausal women with arterial hypertension were evaluated before, immediately after, 1 and 24h after 3 situations: control, RT performed with 6 or 15RM | HR was higher for 15RM than 6RM and control immediately after and 1 h after session. RPP values for 15RM were significantly higher than 6RM and control immediately after the session and remained higher than control 1 h after session. rMSSD were lower after 15RM than 6RM and control. Parasympathetic activity (HF) decreased and sympathetic (LF) activity increased for 15RM when compared to the 6RM and control session immediately after session. |

COPD – chronic obstructive pulmonary disease

CRP – C reactive protein

DBP – diastolic blood pressure

HR – heart rate

MBP – mean blood pressure

NK – natural killers

PPO - peak power output

RM – repetition maximum

RPP – rate pressure product

RT – resistance training

SBP – systolyic blood pressure

URTI – upper respiratory tract infection

VO2 – oxygen consumption

VO2peak - intensity at which the peak of oxygen consumption were consumed at an incremental test.

WBC – white blood cells
